# Effects of Acute Tryptophan Depletion on Brain Serotonin Function and Concentrations of Dopamine and Norepinephrine in C57BL/6J and BALB/cJ Mice

**DOI:** 10.1371/journal.pone.0035916

**Published:** 2012-05-21

**Authors:** Caroline Sarah Biskup, Cristina L. Sánchez, Andrew Arrant, Amanda E. D. Van Swearingen, Cynthia Kuhn, Florian Daniel Zepf

**Affiliations:** 1 Department of Child and Adolescent Psychiatry, Psychosomatics and Psychotherapy, RWTH Aachen University, Aachen, Nordrhein-Westfalen, Germany; 2 Institute for Neuroscience and Medicine, Jülich Research Centre, Jülich, Nordrhein-Westfalen, Germany; 3 JARA Translational Brain Medicine, Aachen & Jülich, Nordrhein-Westfalen, Germany; 4 Department of Pharmacology and Cancer Biology, Duke University Medical Center, Durham, North Carolina, United States of America; INSERM/CNRS, France

## Abstract

Acute tryptophan depletion (ATD) is a method of lowering brain serotonin (5-HT). Administration of large neutral amino acids (LNAA) limits the transport of endogenous tryptophan (TRP) across the blood brain barrier by competition with other LNAAs and subsequently decreases serotonergic neurotransmission. A recent discussion on the specificity and efficacy of the ATD paradigm for inhibition of central nervous 5-HT has arisen. Moreover, side effects such as vomiting and nausea after intake of amino acids (AA) still limit its use. ATD Moja-De is a revised mixture of AAs which is less nauseating than conventional protocols. It has been used in preliminary clinical studies but its effects on central 5-HT mechanisms and other neurotransmitter systems have not been validated in an animal model. We tested ATD Moja-De (TRP−) in two strains of mice: C57BL/6J, and BALB/cJ, which are reported to have impaired 5-HT synthesis and a more anxious phenotype relative to other strains of mice. ATD Moja-De lowered brain TRP, significantly decreased 5-HT synthesis as indexed by 5-HTP levels after decarboxlyase inhibition, and lowered 5-HT and 5-HIAA in both strains of mice, however more so in C57BL/6J than in BALB/cJ. Dopamine and its metabolites as well as norepinephrine were not affected. A balanced (TRP+) control mixture did not raise 5-HT or 5-HIAA. The present findings suggest that ATD Moja-De effectively and specifically suppresses central serotonergic function. These results also demonstrate a strain- specific effect of ATD Moja-De on anxiety-like behavior.

## Introduction

The neurotransmitter serotonin (5-HT) plays a key role in many physiological processes, including cognition and mood, which are typically affected in clinical depression and anxiety disorder. A well-established method to study the effects of 5-HT in the human brain is to lower the central nervous 5-HT synthesis rate by diminishing the availability of tryptophan (TRP), the amino acid precursor of 5-HT. This technique is called acute tryptophan depletion (ATD). The rate controlling step in central nervous 5-HT synthesis is the conversion of TRP into L-5-hydroxytryptophan (5-HTP) by tryptophan hydroxylase (TPH) [1]. As TPH is not saturated at physiological concentrations of TRP, diminished substrate availability for TPH decreases brain 5-HT synthesis and release [1]. Therefore, serotonergic function can be temporarily suppressed by using ATD, a method that is widely used in psychiatric and pharmacological research [2], [3]. Ingestion of a TRP-free amino acid mixture provides a dose of large neutral amino acids which compete with endogenous TRP for transport across the blood-brain barrier, and subsequently lowers brain TRP levels, 5-HT synthesis and levels of 5-HIAA, the primary metabolite of 5-HT [4], [5], [6].

ATD has been used widely to probe serotonergic function in humans. It can induce relapses in patients in remission from major depression and other psychiatric disorders [3]. It can also lower mood in healthy patients with a family history of depression [7]. Some authors even suggest using ATD as a predictive test for personalized antidepressant treatment [8]. The limiting side effect of ATD in human studies is the marked nausea that the amino acid mixture can cause. A modified mixture, ATD Moja-De, involves a body weight adapted administration of amino acids and lower concentration of methionine relative to conventional mixtures, which makes it less nauseating in humans. Its use has proven to be a safe and effective method of TRP depletion even in children and adolescents [9], [10], [11], [12], [13], [14], [15]. It has been shown that ATD Moja-De significantly lowers influx of TRP into the brain in humans [16], but its specific effect on brain 5-HT has not yet been established, which was the aim of this study. Another goal of this study was to adapt ATD to mice in order to conduct mechanistic studies of this widely used physiological manipulation.

There is a current debate about the efficacy and specificity of ATD as regards the central nervous 5-HT system. ATD with complex amino acid mixtures has been widely shown to decrease 5-HT synthesis and release in rat models [4], [17], [18], [19]. However, Van Donkelaar et al. [20] state that a TRP balanced control condition (TRP+) has not yet been investigated thoroughly enough to state it is a valid control condition in behavioral studies [20]. In contrast, Crockett et al. [21] argue that the specificity of ATD effects is clearly established [20]. Clinical data show that only vulnerable human subjects show a change in behavior after ATD, which may point to an individual vulnerability [20].

The purpose of the present study was to verify in an animal model that ATD Moja-De, a revised mixture containing a restricted set of amino acids (phenylalanine, leucine, isoleucine, methionine, valine, threonine and lysine) significantly decreases brain TRP and lowers 5-HT synthesis and release and to test the possibility that individuals or populations with vulnerable 5-HT systems are more sensitive to ATD. Numerous animal studies have documented that ATD can be effective in rats [4], [22] but none have documented the efficacy of this treatment in mice. We used mice in the present study in order to validate this model for future studies in genetically manipulated animals. Only one study has reported the effects of ATD in mice [23], but this study did not verify inhibition of 5-HT synthesis. We evaluated 5-HT synthesis by measuring the accumulation of the precursor 5-HTP after decarboxylase inhibition with NSD 1015, and also quantitated 5-HT and 5-HIAA content, as an indirect measure of 5-HT release [24], [25].

To test the effects of ATD Moja-De we used two strains of mice that were predicted to respond differently to ATD based on previously reported behavioral and neurochemical differences. C57BL/6J (C57) mice have been reported to be resistant to the effects of ATD [23] while BALB/cJ mice (BALBc) have a mutation in TPH2 which lowers their baseline 5-HT production [26], [27] and would be predicted to exhibit an exaggerated response to ATD. We hypothesized that ATD Moja-De would significantly lower brain 5-HT synthesis and serotonergic activity, without affecting dopamine or norepinephrine [28] and that BALBc mice would exhibit a larger neurochemical response to ATD than C57 mice.

## Materials and Methods

### ATD Moja-De

The quantities of the Moja-De amino acid mixture lacking TRP (TRP−) were as follows: L-phenylalanine 1.32 g, L-leucine 1.32 g, L-isoleucine 0.84 g, L-methionine 0.5 g, L-valine 0.96 g, L-threonine 0.6 g, L-lysine 0.96 g (quantities refer to a dose in humans per 10 kg body weight). For the control balanced amino acid mixture (TRP+), 0.7 g of L-TRP (per 10 kg body weight corresponding to an administration in humans) were added to the mix. The mixture was suspended in deionized water at a concentration of 0.2 g/mL using a polytron and sonication bath. Mice were treated with 2 g/kg BW delivered by oral gavage in two doses of 10 mL/kg BW spaced 30 minutes apart. All amino acid mixtures were prepared by the Pharmacy of University Hospital of RWTH Aachen, Germany.

### Animals

Male BALB/cJ mice and male C57BL/6J mice from Jackson Laboratory (Bar Harbor, Maine, USA) were used. They were given one week to acclimate to Duke University's AALAC -accredited vivarium before the study was initiated. The mice were postnatal day (PD) 75 at the time of euthanasia. All procedures were reviewed and approved by the Animal Care and Use Committee at Duke University Medical Center. Mice were housed in groups of 4 to 6 and maintained on a 12∶12-h light-dark schedule (lights on from 6.00 to 18.00 h), in a temperature-controlled (21±1°C) and air conditioned housing room. Food (LabDiet, USA) and water were available *ad libitum*, except for the nights before testing. Animal cages were provided with nestlets for environmental enrichment. Clean cages were provided weekly. Animals received two tests with the amino acid mixture separated by two weeks. For the present study, they were food deprived overnight, treated by gavage with TRP+, TRP− or water followed by saline or 3-hydroxybenzylhydrazine (NSD1015), and were killed for determination of blood and brain TRP and neurotransmitter levels.

### Time Course

The time point of blood and brain collection was chosen based on one previous study which reported on ATD in mice [23], and a pilot time course in both strains of mice performed here to assess the time course of maximal suppression of brain serotonergic measures. Blood and brain samples were collected at the time of gavage (0 minutes) or 90, 150, 210, 270 or 330 minutes after the first gavage (n = 3–4/group for C57, n = 4–8/group for BALBc). Blood and brains were processed as described below.

### Behavior

The time course of the behavioral experiment is shown in Table 1. Anxiety-like behavior was measured using the Light/Dark-Test 2.5 hours after a treatment with the TRP+ mixture, TRP− or water. The test apparatus consisted of a clear Plexiglas box lit at 450 lux and a dark Plexiglas insert. Both compartments (40×40×20 cm) were connected by an 8.5 by 7.5 cm aperture. Each mouse was placed in the dark compartment with the aperture closed. The automatic recording was started as soon as the door was lifted. Percent time spent in light, percent distance traveled in light, latency to emerge into the light, entries into light, total ambulations and pokes into light were automatically recorded using an infrared beam frame and the Hamilton Kinder Motor Monitor software.

**Table 1:** Time Course of Behavioral Experiment.

Time (min)	
Overnight (4.30 p.m. until 9 a.m.)	Food deprivation with water ad libitum
t = 0	First dose of TRP−, TRP+ (2 g/kg) or water (10 ml/kg BW)
t = 30	Second dose of TRP−, TRP+ (2 g/kg) or water (10 ml/kg BW)
t = 150	Testing in Light/Dark-Box

### Blood samples and brain tissue

The time course of the neurochemistry experiment is outlined in Table 2 and was as follows: at t = 0, the first dose of TRP+, TRP− or water was administered by gavage and repeated at t = 30 min. Two hours after the first dose of amino acids or water, the animals were injected i.p. with saline (1 µl/g BW) or the decarboxylase inhibitor NSD-1015 (100 mg/kg BW in saline, 1 µl/g BW) to inhibit TPH2. Measuring the accumulation of 5-HTP after decarboxylase inhibition provides an estimate of TPH activity [29]. Mice were anesthetized with isoflurane 150 min after the first dose and blood was collected via cardiac puncture. Animals were decapitated, brains collected and immediately dissected on ice. Brains were sectioned in a brain block, and brain areas dissected from 1 mm brain slices based on a mouse brain atlas. Prefrontal cortex, frontal cortex and hippocampus were collected bilaterally, immediately weighed and subsequently frozen on dry ice. Prefrontal cortex was taken from slices of the most frontal part of the brain, which included only cortex and olfactory bulb. The frontal cortex was set to be the cortex over the most frontal part of the corpus callosum. Brain tissue was stored at −80°C until assay. The average weight of PFC tissue in BALBc mice was 10.8±2.7 mg, in C57 mice it was 10.3±3.1 mg; the FC tissue weighed 10.1±1.5 mg on average in BALBc mice and 11.1±2.2 mg on average in C57 mice; the hippocampus tissue collected weighed 10.9±2.3 mg in BALBc mice and 10.5±2.0 mg in C57 mice.

**Table 2:** Time Course of Biochemistry Experiment.

Time (min)	
Overnight (4.30 p.m. until 9 a.m.)	Food deprivation with water ad libitum
t = 0	First dose of TRP+, TRP− or water (10 ml/kg BW)
t = 30	Second dose of TRP+, TRP− or water(10 ml/kg BW)
t = 120	Injection with NSD1015 or vehicle
t = 150	Blood draw via cardiac stick, then decapitation

### Biochemistry

Blood for assessment of TRP content was collected in Eppendorf vials and kept on ice until centrifuged (16,000×g for 20 minutes at 4° Celsius) in order to separate the plasma. Plasma samples were stored at −80°C. For brain TRP, 5-HTP, 5-HT, 5-HIAA, norepinephrine (NE) and dopamine (DA) determination, 250 µl of an ice-cold buffer (0.5 mM sodium metabisulfate, 0.2N perchloric acid and 0.5 mM EDTA) was added to the tissue. The tissue was then homogenized by sonication. After a 10 minute spin (16,000×g at 4°C) the supernatant was kept on ice until analysis. The aliquot was used for two separate analyses of 5-HTP, monoamines and their metabolites and for TRP-levels.

Brain neurotransmitter and metabolite content and 5 HTP levels were quantified using a reverse phase high-performance liquid chromatography (RP-HPLC) system with electrochemical detection. The mobile phase for tissue content determination contained 0.1 M sodium phosphate, 0.8 mM octanesulphonic acid, 0.1 mM Na_2_EDTA and 18% methanol; the pH was adjusted to 3.10 using hydrochloric acid and the flow rate was 0.7 ml/min. Samples were quantified with a BAS Epsilon Electrochemical Detector with dual 3 mm glassy carbon electrode (MF-1000) set to 0.70V.

A separate RP-HPLC system was used to measure TRP. The mobile phase included 0.05 M citric acid, 0.05 M sodium phosphate, 0.1 mM Na_2_EDTA and 8% acetonitrile; the pH was not adjusted, and the flow rate was 1 ml/min. Samples were quantitated with a BAS LC-4B detector set to 0.85V.

An external standard curve of all compounds was run on each analysis day. The concentrations for total plasma TRP were expressed in µg/ml plasma. The amount of brain TRP was expressed as µg/mg tissue. The amount of 5-HT, DA and their precursors and metabolites were expressed as ng/mg tissue.

### Statistics

Mean and SEM concentrations were calculated for each treatment group separately. Each dependent measure was analyzed for all groups (water, TRP−, TRP+) using a global 4-way ANOVA repeated measures analysis of variance (ANOVA) with between factors strain, treatment and NSD and region as a within factor (Table S1). Lower level ANOVAs were then conducted to compare the TRP− group to the TRP+ group to the optimal test of the effects of ATD. Data from animals treated with water alone were also analyzed using a repeated measures ANOVA with the same factors but disregarding the treatment as a between factor to identify strain differences in the baseline values and response to NSD1015. Additional details about lower level ANOVAs are provided in supplementary online materials (Materials S1).

A post-hoc Fisher least protected significant difference test (pLSD) was used to identify differences between specific treatment groups. Outliers in biochemical data were identified by means of GRUBBS. In all cases the level of statistical significance was set at p<0.05. Analyses were performed using the Number Cruncher Statistical System (NCSS) for Windows. Graphs were drawn using Graph Pad Prism, version 5 for Windows (GraphPad Software, San Diego, California, USA).

## Results

### Time Course

**Figure 1:**
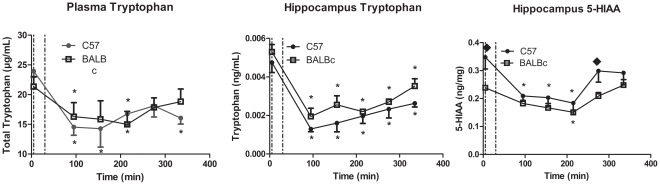
Time course of ATD Moja-De in both strains. (a) Plasma = plasma tryptophan (µg/ml plasma) (b) HPC TRP = tryptophan in hippocampus (µg/mg tissue) (c) HPC-5-HIAA = 5-HIAA in hippocampus (ng/mg tissue). N = 3–4/group for C57, N = 4–8/group for BALBc. * different from Time 0 and ♦ different from corresponding group in BALBc mice.

Data for plasma and hippocampus are shown in Figure 1. Data for other brain areas were comparable. Dotted lines indicate times at which TRP− was administered. Two-way ANOVA of plasma TRP yielded a significant effect of time (F (5,40) = 3.44, p<.012). Post-hoc tests showed that time 0 was different from all other times. For hippocampal TRP, ANOVA indicated a significant effect of both strain (F (5,34) = 8.41, p<.006) and time (F (5,34) = 23.81, p<.000001). Post-hoc tests showed that under depletion hippocampal TRP in BALBc mice was slightly higher than in C57 mice especially at later time points, although plasma TRP was the same for both strains. Time 0 was different from all other times. Finally, for 5-HIAA, ANOVA yielded a significant effect of strain (F (5,40) = 11.96, p<.0014) and time (F (5,40) = 6.54, p<.0002). Post-hoc tests showed that 5-HIAA was lower in BALBc than in C57 mice and that time 0 was different from time 90, 150 and 210. 5-HIAA was decreased more consistently in C57 mice than in BALBc mice. This study showed that plasma and hippocampal TRP remained suppressed for the entire time course, but that 5-HIAA was decreased only at the later times. Subsequent behavioral and biochemical studies were conducted at 150 minutes, when effects on brain serotonergic function were expected to be maximal.

### Tryptophan

**Figure 2:**
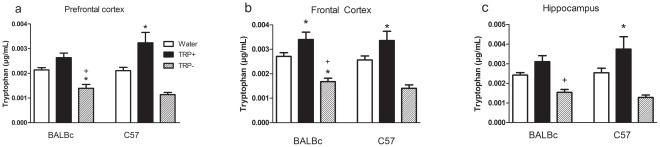
Brain tryptophan. Brain tryptophan content in µg/mg tissue (a) prefrontal cortex (b) frontal cortex and (c) hippocampus. BALBc and C57 mice received two treatments by gavage of TRP+, TRP− mixtures or water vehicle at 30 minute intervals followed by saline or NSD 1015 to inhibit amion acid decarboxylase at 2 hours. Animals were killed 2.5 hours after the first treatment. Data are collapsed for NSD treatment as no significant effects were predicted or observed. N = 11–12/group. * different from Water. +different from TRP+, ♦ different from corresponding group in BALBc mice. TRP+ and TRP− effectively raised and lowered TRP respectively, although the depletion condition was more consistently effective than the control condition. Similar effects were detected in both strains.

The effect of the amino acid mixtures on TRP relative to each other and water are shown in Figure 2 (*a, b, c*) (the data have been collapsed for NSD 1015 treatment since no effect was either observed or expected). All data from both vehicle and NSD-treated animals and details of the statistical results (lower level ANOVAs, interactions, p and F values) are available as supplementary online materials. TRP+ increased TRP relative to water in all brain regions and strains, and TRP− lowered TRP comparably in all brain regions and strains relative to water (main effect of treatment by ANOVA). Post-hoc tests showed that TRP content of prefrontal cortex was lower than frontal cortex and hippocampus. Overall, the results show that TRP+ and TRP− amino acid mixtures effectively raised and lowered TRP in the brain respectively, although the depletion condition was more consistently effective than the control condition. The effects of the TRP manipulations were similar in both the BALBc and C57 strains.

### 5-HTP

**Figure 3:**
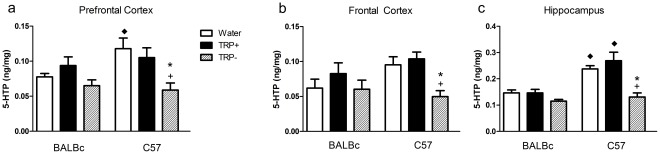
Brain 5-HTP. Brain 5-HTP content in ng/mg tissue in (a) prefrontal cortex (b) frontal cortex and (c) hippocampus. Animals were treated as described in Figure 2. Only NSD 1015-treated animals are shown. N = 6/group. * different from Water, +different from TRP+, ♦ different from corresponding group in BALBc mice. TRP− decreased 5-HT synthesis in all brain regions, but the results were only statistically relevant in C57 mice; TRP+ did not increase 5-HT synthesis; at baseline, BALBc mice had a lower level of 5-HT synthesis in the hippocampus and the prefrontal cortex, but not in the frontal cortex.

The effects of amino acid mixtures on 5-HTP relative to each other and water after the administration of NSD 1015 are shown in Figure 3 (*a, b, c*). This figure shows only values after NSD 1015, as basal 5-HTP was at the limit of detection, as was expected. All values from both saline and NSD-treated animals are available as supplementary online material (Table S2). The ANOVA showed main effects of strain, treatment, and region and interactions of strain, treatment and region. TRP− treatment differed from both TRP+ and water, BALBc mice differed from C57 mice and hippocampus varied from other regions. Lower level ANOVAs and post-hoc analyses showed that: (1) TRP− decreases 5-HT synthesis in all assessed brain regions of C57 mice but not BALBc mice as revealed by a treatment by strain interaction, (2) TRP+ did not increase 5-HT synthesis as shown by a lack of an effect of treatment on synthesis, and (3) at baseline, BALBc mice have a lower level of 5-HT synthesis in the hippocampus and the prefrontal cortex, but not in the frontal cortex as shown by a strain by region interaction.

### 5-HT

**Figure 4:**
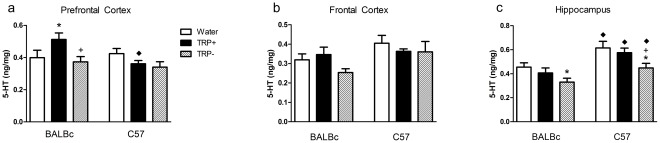
Brain 5-HT. Brain 5-HT content in ng/mg tissue in (a) prefrontal cortex (b) frontal cortex and (c) hippocampus. Animals were treated as described in Figure 2. Only vehicle treated animals are shown. N = 5–6/group. * different from Water, +different from TRP+, ♦ different from corresponding group in BALBc mice. 5-HT content was lower at baseline in BALBc in the hippocampus; TRP− significantly decreased 5-HT content in the hippocampus; TRP+ did not consistently increase 5-HT content; TRP− was consistently lower than TRP+ in all regions.

The effects of the amino acid mixtures on 5-HT relative to each other and water in vehicle-treated animals are shown in Figure 4 (*a,b,c*). All values from both saline and NSD-treated animals are available as supplementary online materials. In summary, the 5-HT findings show that (1) ANOVA showed main effects of strain, treatment (TRP− different from TRP+ and water) and region as well as interactions of strain by region. Post-hoc tests showed that (1) 5-HT content was lower at baseline in BALBc relative to C57 mice in the hippocampus, (2) TRP− significantly decreased 5-HT content relative to TRP+ in the hippocampus, but (3) TRP+ did not consistently increase 5-HT content.

### 5-HIAA

**Figure 5:**
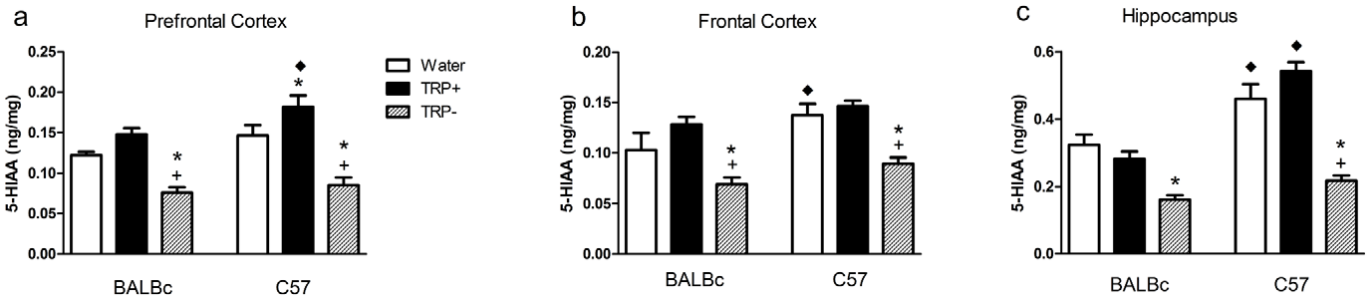
Brain 5-HIAA. Brain 5-HIAA content in ng/mg tissue in (a) prefrontal cortex (b) frontal cortex and (c) hippocampus. Animals were treated as described in Figure 2. Only vehicle treated animals are shown. N = 5–6/group. * different from Water, +different from TRP+, ♦ different from corresponding group in BALBc mice. 5-HIAA was decreased by TRP− in every brain region; a strain difference in 5-HIAA levels at baseline was seen in hippocampus and frontal cortex, but not in the prefrontal cortex.

The effect of the amino acid mixtures on 5-HIAA relative to water and each other in vehicle-treated animals are shown in Figure 5 (*a, b, c*). To summarize these overall findings it can be said that (1) ANOVA showed significant effects of strain, treatment, region and interactions of strain by region, treatment by region, and their three-way interaction (strain by treatment by region). (2) 5-HIAA was decreased by TRP− relative to TRP+ in every brain region investigated and (3) 5-HIAA levels at baseline in BALBc mice were lower than C57 mice in hippocampus and frontal cortex, but not in the prefrontal cortex.

### Other monoamines

DA, DOPAC and HVA did not show any strain differences (p = .36, p = .53 and p = .50 respectively, data not shown) or effects of treatment. Norepinephrine was not affected by any treatment but was significantly lower in BALBc than C57 mice, which held true for all treatment groups (water: F (1,20) = 27.09, p<.00005). All values are available as supplementary online material.

### Behavior

**Figure 6:**
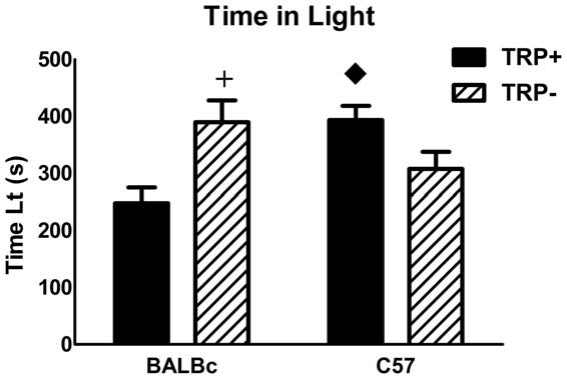
Behavioral data showing time spent in the light. Only TRP+- and TRP− -treated animals are shown. N = 14/group. +different from TRP+, ♦ different from corresponding group in BALBc mice.

The effects of ATD on anxiety-like behavior (time in light) are shown in Figure 6. Time in light and percent time in light yielded similar results. None of the other parameters were different and are not shown. Two-way ANOVA showed that the strains responded differently to TRP− (F (1,52) = 15.25, p<.0003 for strain by treatment interaction). BALBc mice spent significantly less time in the light than C57 mice, but after TRP− BALBc mice increased the time spent in light while C57 mice were not affected. These data suggest that TRP− was anxiolytic in BALBc but not C57 mice.

## Discussion

The main finding of this study is that the treatment with ATD Moja-De (TRP−) decreased brain TRP and subsequently brain 5-HT synthesis as shown by 5-HTP after decarboxylase inhibition and by decreases in 5-HT and 5-HIAA levels of both strains of mice relative to TRP+. The treatment was most effective in the hippocampus, but also decreased serotonergic function in frontal and prefrontal cortex. TRP− decreased serotonergic function more in C57 than in BALBc mice, in contrast to our prediction. ATD did not affect dopamine, its metabolites or norepinephrine in either of the two strains.

Although the ability of ATD to decrease 5-HT synthesis and content is well established in rats and in humans [1], [2], [8], one report in mice yielded equivocal results [23]. This may be due to the differences in amino acid mixtures, since the other study used a TRP-free protein-carbohydrate nutritional mixture. This mixture contained more amino acids than Moja-De, which reduces the fraction of TRP in the control mixture. That mixture was also not administered based on body weight. The present results show that 5-HT synthesis was decreased, and that 5-HIAA levels decreased more than 5-HT levels. These data suggest that 5-HT release was reduced by this treatment. Although it has been proposed that ATD might influence MAO activity [23], such an effect would not explain the present results, as it would result in a concomitant decrease in 5-HIAA and increase in 5-HT. Numerous (but not all) rodent studies support the ability of ATD to decrease 5-HT content [18], [30]. The present results provide at least indirect support for the latter finding that ATD transiently lowers serotonergic function in the brain.

The effects of ATD were substantially greater in the hippocampus than in cortical regions. There are several possible explanations for this. First, it has been shown in mice and rats that 5-HT turnover varies by region [31], [32]. This is likely due to varying levels of afferent input for the specific raphe cell groups that project to different areas as well as varying levels of autoreceptor inhibition of cell firing. Studies with 5-HT1a agonists and antagonists show that these effects are greatest in hippocampus, which might explain the more rapid response to synthesis inhibition [33], [34].

The deficient 5-HT synthesis in BALBc mice relative to other strains was confirmed in this study. 5-HT synthesis and content have been reported to be lower in BALBc mice than in strains without a TPH2 mutation [26], [27]. In the present study, 5-HT synthesis was lower in BALBc than C57 mice, but 5-HT content was lower only in hippocampus. This regional specificity has been reported elsewhere [26], and may reflect strain-dependent adaptations to the lower rate of 5-HT synthesis. In addition, the affinity of this mutant form of the enzyme TPH2 has a higher affinity for the substrate TRP [35], which could offset its lower Vmax, especially under conditions of lower TRP availability. Another possible explanation for the smaller strain difference in the present study could be the food deprivation prior to testing or differences in the time of day at sampling as TRP levels vary significantly over the course of a day, mainly due to the timing of meals [36].

The balanced amino acid mixture (TRP+) increased TRP levels, but did not enhance 5-HT synthesis consistently. This was unexpected, in particular because TPH2 is not saturated with TRP under baseline conditions and due to animals having been food deprived [37], [38]. However, such studies have not been conducted in mice. The TRP+ treatment did cause an increase of 5-HT content in BALBc mice, as predicted based on their lower levels of 5-HT synthesis at baseline. The finding that TRP+ did not consistently enhance 5-HT synthesis is of particular importance for human studies, as the latter employ the same amino acid formulations as a control condition.

The depletion paradigm was more effective in C57 mice than in BALBc mice, which contradicts our hypothesis. We predicted that BALBc mice would be more affected by ATD since their 5-HT synthesis is slowed by a TPH2 mutation [39]. There are several possible explanations for this outcome. First, this study only looked at one time point. However, the time course showed comparable TRP depletion in both strains. More plausibly, the increased affinity for TRP [35] exhibited by this mutation might render it less sensitive to physiologic variation in TRP availability. BALBc mice might also have developed a mechanism to compensate for the lifelong decrease in TPH2 function. Alternatively, there might be a threshold level below which 5-HT content cannot be decreased with dietary manipulations. As BALBc mice are closer to the threshold at baseline, they would reach this “floor effect” faster.

We found that BALBc mice have significantly lower norepinephrine levels in all brain regions compared to C57 mice. This could be a supplementary explanation for the anxious phenotype of BALBc mice reported previously, especially since additional impairment of 5-HT function by TRP depletion relieved rather than exacerbated anxiety [40], [41]. A slight (15%) difference in norepinephrine content between these two strains has been reported previously [42], [43]. The larger difference reported here may be due to starvation, but could also be an effect of strain differences that have emerged since the studies were published.

Behavioral results showed strain selectivity in the effects of ATD Moja-De on anxiety-like behavior. At baseline, BALBc are more anxious then C57 mice, as one would expect based on previous studies with this strain [44], [45]. However, impairment of serotonergic function has an anxiolytic effect on BALBc, but not on C57 mice, suggesting the BALBc mice have an increased vulnerability towards a 5-HT imbalance while mice without a TPH2 mutation can compensate for the impairment in synthesis. The directionality of the behavioral effects was unexpected, as we predicted 5-HT depletion would have worsened anxiety-like behavior in BALBc mice given their baseline 5-HT deficit. Adaptations to the lifelong reduction in 5-HT synthesis might have contributed to the observed responses after acute manipulations. Alternatively, the behavioral results could reflect behavioral disinhibition in a threatening situation, which is relieved by lowering serotonergic function [46], [47]. The latter scenario would predict greater effects of ATD in the vulnerable (BALBc) genotype. Future experiments will be necessary to resolve these two possibilities.

In summary, the major finding of this study was that ATD Moja-De effectively impaired 5-HT synthesis and lowered 5-HIAA content (an indirect measure of 5-HT release) in mice. The establishment of this paradigm provides a model with which to study the effects of mild serotonergic impairment in genetically manipulated animals. Moreover, the present study suggested that the TRP+ condition may not alter brain 5-HT synthesis, which could make it a valid control condition for studies in humans. The present results show that ATD Moja-De did not affect dopamine, its metabolites or norepinephrine. The data of the present study strongly support the conclusion that ATD Moja-De significantly decreases central serotonergic function in mice and that this decrease is specific for 5-HT relative to other monoaminergic systems.

## Supporting Information

Table S1 4-way ANOVA.
(PDF)

Table S2 TRP and Neurotransmitter Levels.
(PDF)

Materials S1 Statistics and results.
(PDF)
