# Supplementary material for: Effects of Acute Tryptophan Depletion on Brain Serotonin Function and Concentrations of Dopamine and Norepinephrine in C57BL/6J and BALB/cJ Mice
Source: PLoS One. 2012 May 21;7(5):e35916. doi: 10.1371/journal.pone.0035916 (PMC3357407; doi:10.1371/journal.pone.0035916)
Supplement: Table S1 — 4-way ANOVA. (PDF) [file pone.0035916.s001.pdf]

Supplementary Online Material:

Table S1: 4-WAY ANOVA									
**Factor**	**5-HTP**			**5-HT**			**5-HIAA**		
	**df**	**F**	**P**	**df**	**F**	**P**	**df**	**F**	**P**
Strain	1,60	9.03	< .004	1,60	6.06	<.02	1,60	61.80	<.000001
Treatment (TRP+, TRP-)	2,60	9.87	<.0002	2,60	18.32	<.000001	2,60	96.92	<.000001
NSD	1,60	420.1	<.000001	[redacted]			1,60	125.79	<.000001
Region	2,115	148.8	<.000001	2,112	24.90	<.000001	2,118	720.45	<.000001
Strain x Treatment	2,60	2.93	.061	2,60	3.54	<.04	2,60	4.96	<.02
Strain x NSD	1,60	17.22	<.0002	[redacted]			1,60	5.52	<.03
Strain x Region	2,115	21.57	<.000001	2,112	12.86	<.00001	2,118	61.27	<.000001
Treatment x NSD	2,60	12.07	<.00004	[redacted]			2,60	3.00	.057
Treatment x Region	4,115	5.26	<.0007	4,112	1.26	.288	4,118	42.24	<.000001
NSD x Region	4,115	163.57	<.00001	[redacted]			2,118	31.31	<.000001
**Factor**	**Tryptophan**			**NE**			**DA**		
	**df**	**F**	**P**	**df**	**F**	**P**	**df**	**F**	**P**
Strain	1,60	0.05	0.828	1,60	96.54	<.000001	1,60	5.22	<.03
Treatment (TRP+, TRP-)	2,60	31.34	<.000001	2,60	.20	.820	2,60	1.61	.327
NSD	1,60	1.14	.291	1,60	1.30	.259	1,60	8.35	<.006
Region	2,117	12.59	<.00002	2,118	1.91	.152	2,114	96.18	<.000001
Strain x Treatment	2,60	1.07	.349	2,60	.05	.952	2,60	1.71	.190
Strain x NSD	1,60	0.25	.621	1,60	.26	.612	1,60	0.84	.362
Strain x Region	2,117	1.95	.147	2,118	.58	.559	2,114	4.56	.012
Treatment x NSD	2,60	0.09	.911	2,50	0.00	.999	2,60	0.04	.956
Treatment x Region	4,117	0.83	.508	4,118	1.12	.348	4,114	1.59	.181
NSD x Region	4,117	0.15	.861	2,118	1.93	.149	2,114	1.61	.205
**Factor**	**DOPAC**			**HVA**					
	**df**	**F**	**P**	**df**	**F**	**P**			
Strain	1,60	7.43	<.009	1,60	8.30	<.006			
Treatment (TRP+, TRP-)	2,60	1.73	.186	2,60	2.11	.130			
NSD	1,60	46.73	<.000001	1,60	1.35	.250			
Region	2,116	146.19	<.000001	2,116	275.84	<.000001			
Strain x Treatment	2,60	2.87	.065	2,60	2.14	.126			
Strain x NSD	1,60	1.77	.188	1,60	0.21	.649			
Strain x Region	2,116	1.83	.165	2,116	3.12	<.05			
Treatment x NSD	2,60	0.03	.970	2,60	0.01	.991			
Treatment x Region	4,116	1.44	.226	4,116	2.11	.085			
NSD x Region	4,116	25.47	<.000001	2,116	1.40	.252			
