# Supplementary material for: Effects of Acute Tryptophan Depletion on Brain Serotonin Function and Concentrations of Dopamine and Norepinephrine in C57BL/6J and BALB/cJ Mice
Source: PLoS One. 2012 May 21;7(5):e35916. doi: 10.1371/journal.pone.0035916 (PMC3357407; doi:10.1371/journal.pone.0035916)
Supplement: Table S2 — TRP and Neurotransmitter Levels. (PDF) [file pone.0035916.s002.pdf]

Supplementary Online Material:

Table S2
TRP and Neurotransmitter Levels

PFC

Strain	Treat.	TRP (ng/mg tissue)	N	NE (ng/mg tissue)	N	DA (ng/mg tissue)	N	DOPAC (ng/mg tissue)	N	HVA (ng/mg tissue)	N
BALBc	Water	2.14 ± 0.09	12	0.14 ± 0.01	12	0.058 ± 0.010	12	0.021 ± 0.004	12	0.13 ± 0.02	12
	TRP+	2.63 ± 0.18	11	0.15 ± 0.02	12	0.078 ± 0.017	12	0.028 ± 0.006	12	0.16 ±0.02	12
	TRP-	1.39 ± 0.15	12	0.16 ± 0.01	12	0.049 ± 0.008	12	0.021 ± 0.004	12	0.13 ± 0.01	12
C57	Water	2.11 ± 0.13	12	0.23 ± 0.01	12	0.027 ± 0.005	10	0.018 ± 0.004	12	0.11 ± 0.01	12
	TRP+	3.24 ± 0.42	12	0.25 ± 0.02	12	0.030 ± 0.006	12	0.013 ± 0.003	12	0.098 ± 0.007	12
	TRP-	1.14 ± 0.09	12	0.25 ± 0.02	12	0.040 ± 0.006	12	0.015 ± 0.002	12	0.10 ± 0.01	12

FC

Strain	Treat	TRP (ng/mg tissue)	N	NE (ng/mg tissue)	N	DA (ng/mg tissue)	N	DOPAC (ng/mg tissue)	N	HVA (ng/mg tissue)	N
BALBc	Water	2.71 ± 0.15	12	0.14 ± 0.02	12	0.09 ± 0.01	12	0.026 ± 0.005	12	0.14 ± 0.01	12
	TRP+	3.40 ± 0.30	11	0.15 ± 0.01	12	0.11 ± 0.02	12	0.041 ± 0.005	12	0.18 ± 0.02	12
	TRP-	1.68 ± 0.14	12	0.14 ± 0.01	12	0.066 ± 0.008	11	0.023 ± 0.005	11	0.121 ± 0.009	11
C57	Water	2.57 ± 0.17	12	0.23 ± 0.01	12	0.10 ± 0.02	11	0.029 ± 0.005	12	0.143 ± 0.009	12
	TRP+	3.36 ± 0.38	12	0.24 ± 0.01	12	0.09 ± 0.01	12	0.025 ± 0.003	12	0.134 ± 0.007	12
	TRP-	1.41 ± 0.14	12	0.26 ± 0.01	12	0.08 ± 0.01	12	0.025 ± 0.004	12	0.119 ± 0.009	12

HPC

Strain	Treat	TRP (ng/mg tissue)	N	NE (ng/mg tissue)	N	DA (ng/mg tissue)	N	DOPAC (ng/mg tissue)	N	HVA (ng/mg tissue)	N
BALBc	Water	2.42±0.12	11	0.16 ± 0.02	12	0.018 ± 0.003	12	0.006 ± 0.002	12	0.033 ± 0.004	12
	TRP+	3.11±0.31	12	0.14 ± 0.01	12	0.025 ± 0.005	12	0.012 ± 0.003	12	0.042 ± 0.005	12
	TRP-	1.55±0.14	12	0.15 ± 0.02	12	0.023 ± 0.004	12	0.007 ± 0.001	12	0.038 ± 0.005	12
C57	Water	2.54±0.23	12	0.26 ± 0.02	12	0.014 ± 0.003	12	0.003 ± 0.001	12	0.024 ± 0.003	12
	TRP+	3.74 ±0.64	12	0.26 ± 0.01	10	0.015 ± 0.002	11	0.004 ± 0.001	11	0.027 ± 0.002	10
	TRP-	1.28 ±0.12	12	0.25 ± 0.02	12	0.015 ± 0.003	11	0.004 ± 0.001	11	0.028 ± 0.003	11

(Table S2 continued)

PFC								
**Strain**	**Treatment**	**NSD1015**	**5-HTP (ng/mg tissue)**	**N**	**5-HT (ng/mg tissue)**	**N**	**5-HIAA (ng/mg tissue)**	**N**
BALBc	Water	yes	0.078 ± 0.005	6	0.41 ± 0.02	6	0.073 ± 0.010	6
		no	0.018 ± 0.010	6	0.40 ± 0.07	5	0.122 ± 0.004	6
	TRP+	yes	0.094 ± 0.012	6	0.49 ± 0.04	6	0.104 ± 0.013	6
		no	0.007 ± 0.002	5	0.51 ± 0.04	6	0.148 ± 0.008	6
	TRP-	yes	0.065 ± 0.008	6	0.38 ± 0.03	6	0.041 ± 0.003	6
		no	0.014 ± 0.005	5	0.37 ± 0.03	6	0.076 ± 0.006	6
C57	Water	yes	0.118 ± 0.015	6	0.46 ± 0.03	6	0.095 ± 0.007	6
		no	0.006 ± 0.004	6	0.42 ± 0.03	6	0.146 ± 0.013	6
	TRP+	yes	0.105 ± 0.014	6	0.43 ± 0.03	6	0.094 ± 0.011	6
		no	0.004 ± 0.001	5	0.36 ± 0.02	5	0.182 ± 0.014	6
	TRP-	yes	0.059 ± 0.010	6	0.31 ± 0.02	6	0.051 ± 0.005	6
		no	0.006 ± 0.003	6	0.34 ± 0.03	5	0.085 ± 0.009	6
**FC**								
**Strain**	**Treatment**	**NSD1015**	**5-HTP (ng/mg tissue)**	**N**	**5-HT (ng/mg tissue)**	**N**	**5-HIAA (ng/mg tissue)**	**N**
BALBc	Water	yes	0.062 ± 0.013	6	0.28 ± 0.03	6	0.052 ± 0.007	6
		no	0.015 ± 0.006	6	0.32 ± 0.03	6	0.103 ± 0.017	6
	TRP+	yes	0.083 ± 0.015	6	0.54 ± 0.15	6	0.092 ± 0.019	6
		no	0.017 ± 0.005	6	0.35 ± 0.04	6	0.128 ± 0.007	6
	TRP-	yes	0.060 ± 0.013	6	0.21 ± 0.02	6	0.036 ± 0.006	6
		no	0.021 ± 0.007	6	0.25 ± 0.02	6	0.069 ± 0.007	6
C57	Water	yes	0.095 ±0.011	6	0.36 ± 0.02	5	0.081 ± 0.006	6
		no	0.005 ± 0.004	5	0.40 ± 0.04	5	0.137 ± 0.011	6
	TRP+	yes	0.104 ± 0.010	6	0.40 ± 0.03	5	0.094 ± 0.006	6
		no	0.008 ± 0.004	6	0.36 ± 0.01	6	0.146 ± 0.005	6
	TRP-	yes	0.050 ± 0.009	6	0.29 ± 0.03	5	0.043 ± 0.004	6
		no	0.014 ± 0.007	6	0.36 ± 0.05	6	0.090 ± 0.006	6
**HPC**								
**Strain**	**Treatment**	**NSD1015**	**5-HTP (ng/mg tissue)**	**N**	**5-HT (ng/mg tissue)**	N	**5-HIAA (ng/mg tissue)**	**N**
BALBc	Water	yes	0.146 ± 0.011	6	0.39 ± 0.05	6	0.178 ± 0.019	6
		no	0.007 ± 0.002	6	0.45 ± 0.04	6	0.323 ± 0.031	6
	TRP+	yes	0.146 ± 0.014	6	0.47 ± 0.02	6	0.222 ± 0.016	6
		no	0.014 ± 0.004	6	0.41 ± 0.04	6	0.099 ± 0.022	6
	TRP-	yes	0.115 ± 0.003	6	0.32 ± 0.03	6	0.282 ± 0.006	5
		no	0.007 ± 0.003	6	0.33 ± 0.03	6	0.161 ± 0.014	6
C57	Water	yes	0.238 ± 0.012	6	0.53 ± 0.03	6	0.304 ± 0.028	6
		no	0.010 ± 0.003	6	0.61 ± 0.06	6	0.460 ± 0.044	6
	TRP+	yes	0.269 ± 0.032	6	0.54 ± 0.04	6	0.327 ± 0.021	6
		no	0.006 ± 0.003	5	0.58 ± 0.04	5	0.129 ± 0.027	5
	TRP-	yes	0.131 ± 0.016	6	0.39 ± 0.04	6	0.542 ± 0.012	6
		no	0.011 ± 0.006	6	0.45 ± 0.04	6	0.218 ± 0.015	6
