# Supplementary material for: Effects of Acute Tryptophan Depletion on Brain Serotonin Function and Concentrations of Dopamine and Norepinephrine in C57BL/6J and BALB/cJ Mice
Source: PLoS One. 2012 May 21;7(5):e35916. doi: 10.1371/journal.pone.0035916 (PMC3357407; doi:10.1371/journal.pone.0035916)
Supplement: Materials S1 — Statistics and results. (PDF) [file pone.0035916.s003.pdf]

Supplementary Online Material:

Methods: Statistical analysis

For baseline animals, changes in 5-HTP and 5-HIAA were analyzed with a two-way ANOVA with strain and NSD as factors. In lower level ANOVAs, 5-HTP was analyzed in NSD-treated animals only as increases in 5-HTP represent the measure of 5-HT synthesis. As NSD can affect MAO activity [37], lower level ANOVAs were conducted for 5-HT and 5-HIAA on vehicle-treated animals. NSD animals were excluded from 5-HT analysis to be cautious about this dependent variable, even though there was no main effect of NSD in the global ANOVA. Changes in 5-HT, NE and DA were determined by means of a subsequent one-way ANOVA with strain as the between factor. Post-hoc Newman-Keuls-Test was used to identify differences between specific treatment groups. When comparing TRP- to the TRP+ mixture, changes in 5-HTP, 5-HT and 5-HIAA were analyzed with a two-way ANOVA with strain and treatment as factors. Changes in DA and NE were determined by a follow-up two-way ANOVA with strain and treatment as factors because NSD 1015 did not affect DA or NE.

Results: Statistics

Tryptophan

The global ANOVA showed a main effect of treatment ($F(2,60) = 31.34$, $p < .000001$) and region ($F(2,117) = 12.59$, $p < .00002$) but not NSD or strain. Lower level two-way ANOVA of separate brain regions followed by post-hoc tests were used to test for differences between specific treatment groups. For prefrontal cortex, there was a significant effect of treatment ($F(2,65) = 29.86$, $p < .0000001$) but no strain difference. Post-hoc analysis showed that water differed from both TRP+ and TRP- which also differed from each other. However, post-hoc testing showed that for TRP+, TRP concentrations were increased relative to water only in C57 animals. TRP- lowered TRP in both strains relative to water and TRP+.

For frontal cortex, there was a significant effect of treatment ($F(2,65) = 31.87$, $p < .0000001$) and no strain difference. Post-hoc analysis showed that water differed from both TRP+ and TRP- which also differed from each other. TRP+ was increased relative to water in both strains and TRP- was decreased relative to water and TRP+ in both strains.

For hippocampus, there was a significant effect of treatment ($F(2,65) = 19.75$, $p < .000001$). Post-hoc analysis showed that water differed from both TRP+ and TRP- which also differed from each other. Post-hoc tests showed that in C57, TRP+ increased TRP relative to both water and TRP-, and TRP- decreased TRP relative to water. However, in BALBc mice TRP+ and TRP- differed from each other but not from water.

5-HTP

The global ANOVA showed main effects of strain ($F(1,60) = 9.03$, $p < .004$), treatment ($F(2,60) = 9.87$, $p < .0002$), NSD ($F(1,60) = 420.08$, $p < .000001$), region ($F(2,115) = 148.82$, $p < .000001$) and interactions of strain by NSD ($F(1,60) = 17.22$, $p < .0002$), treatment by NSD ($F(2,60) = 12.07$, $p < .12.07$), strain by region ($F(2,115) = 21.57$, $p < .000001$), treatment by region ($F(4,115) = 5.26$, $p < .0007$) and NSD by region ($F(2,115) = 163.57$, $p < .000001$); also, there was a four-way interaction (strain by treatment by NSD by region: $F(4,115) = 3.68$, $p < .008$). Regional differences between the two strains existed, and all regions were affected differently by NSD and treatment with the amino acid mixtures.

Global ANOVA of animals treated with NSD showed a significant effect of strain ($F(1,107) = 14.71$, $p < .0006$), treatment ($F(2,107)= 12.57$, $p < .0001$), region ($F(2,107) = 185.01$, $p < .00001$) and interactions between strain and treatment ($F(2,107) = 3.77$, $p < .034$), strain by region ($F(2,107) = 2011$, $p < .00001$), treatment by region ($F(4,107) = 4.77$, $p < .002$) and a three-way interaction among strain, treatment and region ($F(4,107) = 3.14$, $p < .021$).

Lower level two-way ANOVAs were conducted to analyze the results in different brain regions. In the prefrontal cortex, there was a main effect of treatment ($F(2,30) = 7.00$, $p < .004$). Post-hoc testing showed that 5-HTP after TRP- was lower than water or TRP+. However, TRP+ did not augment 5-HTP levels in the prefrontal cortex compared to water. TRP- differed from both water and TRP+ in C57 mice, but none of the treatment groups differed in BALBc. When comparing TRP+ to TRP-, there was a main effect of treatment ($F(1,20) = 11.03$, $p < .004$), which confirms that the TRP- mixture also decreased 5-HT synthesis relative to animals that received the TRP+ mixture. Post-hoc testing showed that 5-HTP was only decreased in TRP- treated C57 mice, but not in BALBc mice. 5-HTP in water-treated BALBc differed significantly from water-treated C57, indicating that basal 5-HT synthesis was lower in BALBc. In the frontal cortex there was a main effect of treatment ($F(2,30) = 5.13$, $p < .02$). Post-hoc testing revealed that TRP- decreased 5-HTP compared to TRP+ only. The water condition was not different from TRP+ or TRP-. Comparing TRP+ to TRP- showed a main effect of treatment ($F (1,20) = 10.14$, $p < .005$). Post-hoc tests showed that TRP- differed from both water and TRP+ in C57 mice, but not BALBc mice. Baseline 5-HT synthesis did not differ in BALBc mice and C57 mice. In the hippocampus there were main effects of strain ($F(1,30) = 29.32$, $p < .000007$), treatment ($F(2,30) = 13.62$, $p < .00007$) and an interaction of strain by treatment ($F(2,30) = 5.04$, $p < .02$). TRP- differed from both water and TRP+, which only held true for C57 mice. TRP+ did not increase 5-HTP relative to water. When comparing only TRP- and the TRP+, there were main effects of strain ($F(1,20) = 12.59$, $p < .003$), treatment ($F(1,20) = 19.03$, $p < .0004$) and an interaction of strain by treatment ($F(1,20) = 7.53$, $p < .02$). Post-hoc tests showed that TRP- decreased 5-HTP in C57 mice, but not in BALBc mice. 5-HTP in water- and TRP+-treated BALBc differed significantly from water- and TRP+-treated C57 mice, indicating that basal 5-HT synthesis was lower in BALBc mice.

5-HT

Serotonin concentration showed treatment effects, strain differences and regional differences. The global ANOVA revealed a main effect of strain ($F(1,60) = 6.06$, $p < .02$), treatment ($F(2,60) = 18.32$, $p < .00001$), region ($F(2,112) = 24.90$, $p < .000001$), an interaction of strain by treatment ($F(2,60) = 3.54$, $p < .04$) and a strain by region interaction ($F(2,112) = 12.86$, $p < .00001$) but no effect of NSD. BALBc mice had lower 5-HT levels than C57, but both strains were affected by the treatment. A post-hoc test showed that TRP- decreased 5-HT levels compared to water and TRP+. TRP+ increased 5-HT compared to water in BALBc, but not in C57.

A global ANOVA including only vehicle treated animals showed main effects of strain ($F(1,30) = 7.00$, $p < .02$), treatment ($F(2,30) = 6.75$, $p < .004$), region ($F(2,54) = 24.52$, $p < .000001$) and an interaction of strain by region ($F(2,54) = 14.58$, $p < .00001$). TRP- decreased 5-HT content relative to water and TRP+. TRP+ did not augment 5-HT as compared to water. All areas differed from each other. Treatments differed slightly by strain. Post-hoc testing showed that TRP- decreased 5-HT content in the BALBc mice relative to TRP+, but not compared to water. In C57 mice, TRP- lowered 5-HT content compared to water, but not to TRP+.

A lower level two-way ANOVA in separate brain regions followed by post-hoc tests was used to compare specific treatments to each other. In the prefrontal cortex, there were no main effects of strain or treatment. In a lower level two-way ANOVA in the prefrontal cortex comparing TRP+ to TRP- there were main effects of strain ($F(1,18) = 7.33$, $p < .02$) and treatment ($F(1,18) = 5.55$, $p < .03$) which confirms that the treatment with the TRP- mixture decreased 5-HT content compared to TRP+. Post-hoc testing revealed that the treatment with TRP- was only effective in BALBc mice. Baseline 5-HT did not differ between BALBc and C57. In the frontal cortex there was a main effect of strain ($F(1,28) = 6.43$, $p < .02$). BALBc mice had lower 5-HT

than C57 mice. In a lower level two-way ANOVA comparing TRP+ to TRP- in the frontal cortex there were no main effects. In the hippocampus there were main effects of strain ($F(1,29) = 19.50$, $p < .0002$) and treatment ($F(2,29) = 6.73$, $p < .004$). BALBc mice had lower 5-HT content than C57 mice. TRP- lowered 5-HT content relative to water and TRP+. Post-hoc testing showed that TRP- decreased 5-HT content compared to the water condition and in C57 mice also compared to TRP+. However, TRP+ did not increase 5-HT content in the hippocampus compared to the water animals. In a lower level two-way ANOVA comparing TRP+ to TRP-, there were main effects of strain ($F(1,19) = 14.21$, $p < .002$) and treatment ($F(1,19) = 7.16$, $p < .02$). Post-hoc tests showed that in C57 mice TRP- decreased 5-HT content compared to TRP+, but in BALBc the treatment had no effect. Post-hoc tests showed that BALBc mice had lower 5-HT content than C57 mice under all treatment conditions.

5-HIAA

In a global ANOVA for 5-HIAA there were main effects of strain ($F(1,60) = 61.80$, $p < .000001$), treatment ($F(2,60) = 96.92$, $p < .000001$), NSD ($F(1,60) = 125.79$, $p < .000001$), region ($F(2,118) = 720.45$, $p < .000001$), interactions of strain by treatment ($F(2,60) = 4.96$, $p < .02$), strain by NSD ($F(1,60) = 5.52$, $p < .03$), strain by region ($F(2,118) = 61.27$, $p < .000001$), treatment by region ($F(4,118) = 42.24$, $p < .000001$), NSD by region ($F(2,118) = 31.31$, $p < .0000001$), strain by treatment by region ($F(4,118) = 9.53$, $p < .00001$) and strain by NSD by region ($F(2,118) = 3.86$, $p < .03$). BALBc mice had a lower level of 5-HIAA across brain regions. The treatments were effective but to a different extent in both strains and the brain regions investigated. NSD 1015 affected 5-HIAA levels, but to a different extent in both strains and in each respective region.

A global ANOVA including only vehicle treated animals showed main effects of strain ($F(1,30) = 39.54$, $p < .000001$), treatment ($F(2,30) = 50.61$, $p < .000001$), region ($F(2,59) =$

422.03, $p < .000001$) and an interactions of strain by treatment ($F(2,30) = 4.25$, $p < .03$), strain by region ($F(2,59) = 38.43$, $p < .000001$), treatment by region ($F(4,59) = 25.27$, $p < .000001$) and strain by treatment by region ($F(4,59) = 7.48$, $p < .00007$).

Lower level two-way ANOVAs of each brain region were conducted to make specific comparisons. In the prefrontal cortex there were main effects of strain ($F(1,30) = 7.85$, $p < .009$) and treatment ($F(2,30) = 2.65$, $p < .09$). Post-hoc testing showed that BALBc mice had lower levels of 5-HIAA in the prefrontal cortex than C57 mice. TRP+ increased 5-HIAA compared to water and TRP+ and TRP- decreased 5-HIAA relative to both water and TRP+. TRP- decreased 5-HIAA compared to water and TRP+ in both strains. However, TRP+ increased 5-HIAA relative to water only in C57 mice. A lower level two way ANOVA in the prefrontal cortex comparing TRP+ to TRP- showed main effects of strain ($F(1,20) = 4.69$, $p < .05$) and treatment ($F(1,20) = 71.05$, $p < .000001$). Post-hoc testing showed that TRP- decreased 5-HIAA in the prefrontal cortex equally in both strains compared to TRP+. Baseline levels of 5-HIAA were not different between the strains. Similarly, in the frontal cortex there were main effects of strain ($F(1,60) = 10.99$, $p < .002$) and treatment ($F(2,60) = 34.52$, $p < .0000001$). There was no augmentation of 5-HIAA through TRP+ in either strain, however TRP- decreased 5-HIAA relative to TRP+ and water in both strains. A lower level two-way ANOVA in the prefrontal cortex comparing TRP- to TRP+ showed main effects of strain ($F(1,20) = 9.22$, $p < .007$) and treatment ($F(1,20) = 84.11$, $p < .000001$). Post-hoc testing showed that the treatment with TRP- decreased 5-HIAA content in both strains. At baseline 5-HIAA content was lower in BALBc mice compared to C57 mice. In the hippocampus there were main effects of strain ($F(1,29) = 45.43$, $p < .0000001$), treatment ($F(2,29) = 40.58$, $p < .0000001$) as well as interaction of strain by treatment ($F(2,29) = 6.79$, $p < .004$). Post-hoc tests showed that TRP- decreased 5-HIAA compared to water and TRP+, but TRP+ did not increase 5-HIAA. In BALBc mice, TRP- decreased 5-HIAA compared to water, but not TRP+. In C57 mice TRP- decreased 5-HIAA compared to TRP+ and water. In a lower level two-

way ANOVA comparing TRP- to TRP+ there were main effects of strain ($F(1,19) = 65.74$, $p < .002$), treatment ($F(1,19) = 129.59$, $p < .000001$), and a strain by treatment interaction ($F(1,19) = 27.00$, $p < .00006$). Post-hoc testing showed that TRP- decreased 5-HIAA in C57 mice only. The strain by treatment interaction reveals a differential effect of TRP- on 5-HIAA in BALBc mice and C57 mice: in BALBc mice 5-HIAA was decreased less than in C57 mice. At baseline, 5-HIAA content was lower in BALBc mice that C57 mice.
